# Supplementary material for: Dynamical amplification of magnetoresistances and Hall currents up to the THz regime
Source: Sci Rep. 2017 Jun 16;7:3686. doi: 10.1038/s41598-017-03924-1 (PMC5473859; doi:10.1038/s41598-017-03924-1)
Supplement: Supplementary file 1 — Supplementary Information [file 41598_2017_3924_MOESM1_ESM.pdf]

Dynamical amplification of magnetoresistances and Hall currents up to the THz regime

Filipe S. M. Guimarães[1,*], Manuel dos Santos Dias[1], Juba Bouaziz[1], Antonio T. Costa[2], Roberto B. Muniz[2], and Samir Lounis[1]

[1]Peter Grünberg Institut and Institute for Advanced Simulation, Forschungszentrum Jülich & JARA, Jülich D-52425, Germany
[2]Instituto de Física, Universidade Federal Fluminense, Niterói, Brazil
[*]f.guimaraes@fz-juelich.de

SUPPLEMENTARY FIGURES

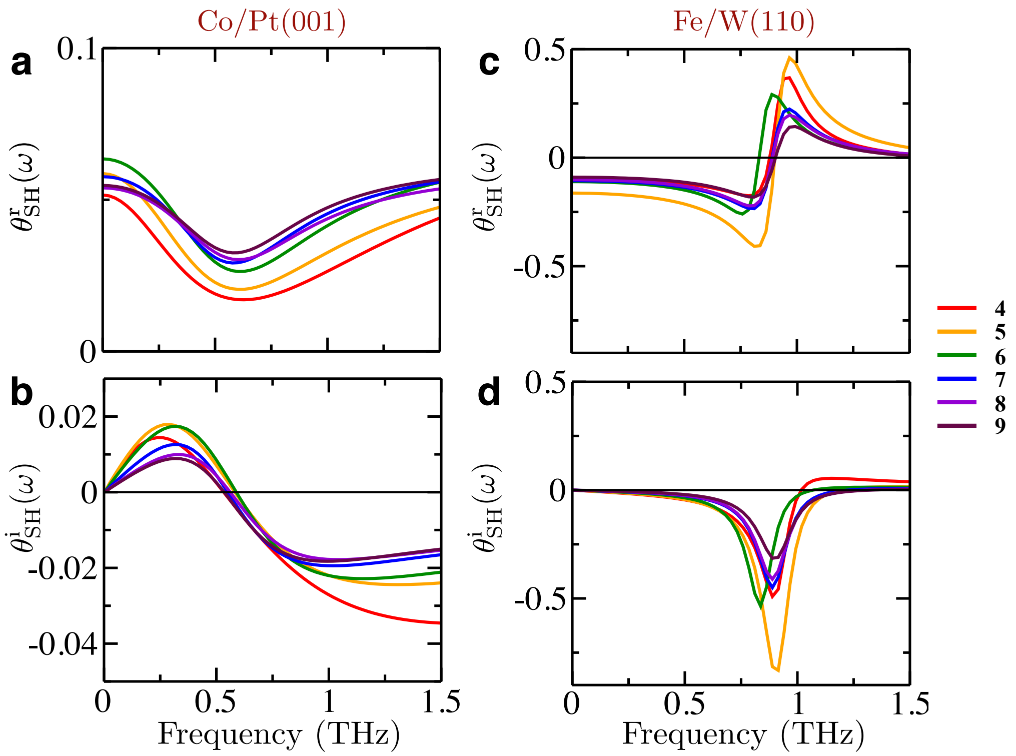

Figure S1. Real and imaginary part of complex spin Hall angle. (a) Real and (b) imaginary part of the complex spin Hall angle calculated for monolayers of Co/Pt(001) as a function of the frequency for different thicknesses of Pt. (c) Real and (d) imaginary part of the complex spin Hall angle calculated for monolayers of Fe/W(110) as a function of the frequency for different thicknesses of W. The substrate thickness, measured in atomic planes, is indicated on the right.

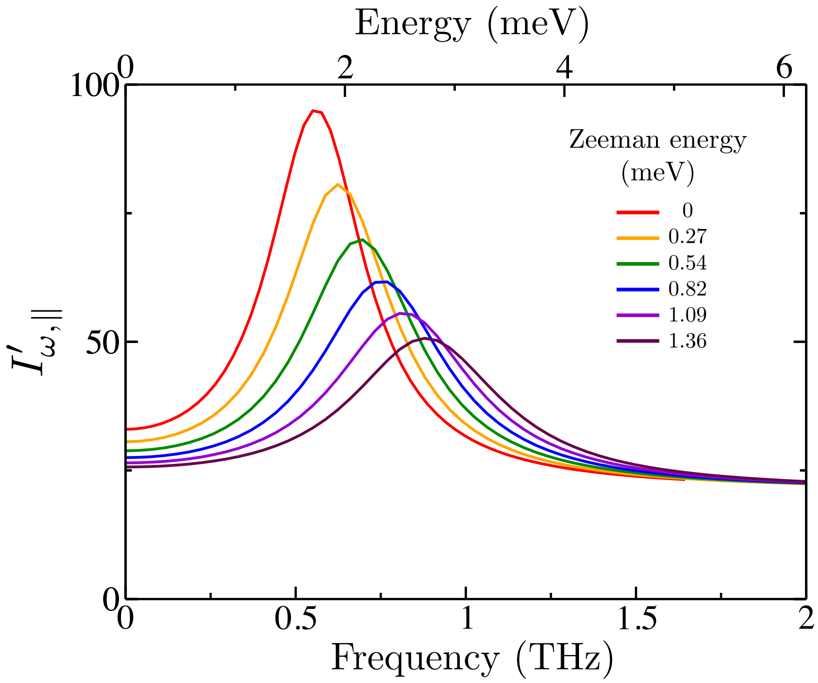

Figure S2. Effect of magnetic field on the dynamical currents. Longitudinal in-phase currents as a function of the frequency for different intensities of the Zeeman energy $g_S \mu_B |\mathbf{B}|$ applied on Co/Pt(001) in the direction of the (out-of-plane) magnetization.

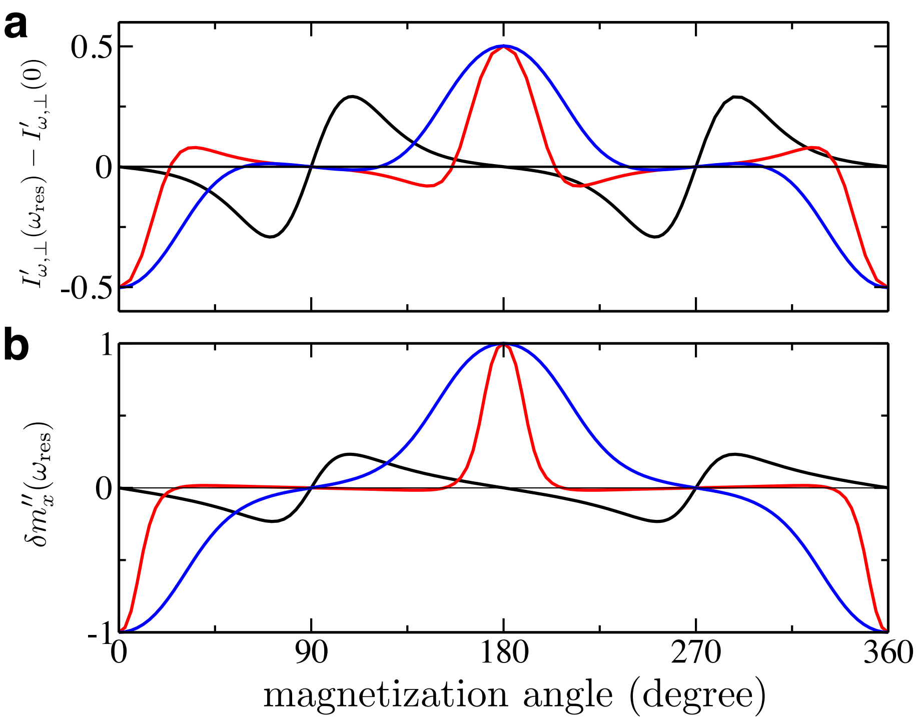

Figure S3. Comparison between dynamical currents and magnetization precession. (a) Dynamical contribution of the transverse charge current flowing on Fe/W(110), obtained by taking the difference between the solid and dashed lines of Fig. 3d of the main text. (b) Normalized out-of-phase component of the projection of the magnetization along the x axis.

SUPPLEMENTARY NOTES

S1 Dynamical conductivity: Phenomenology

The dynamical currents described in the main text is generated by a combination of effects. A common phenomenological approach to describe the currents generated by an oscillatory time-dependent field can be described as follows.

Current-induced magnetization dynamics

The external electric field induces a charge current density $\mathbf{J}_\mathrm{C} = \sigma_0\mathbf{E}$, where σ_0 is the conductivity tensor of the system. On the main text, we calculate the total in-plane charge current flowing through the system, which is given by the integration of the current density over the respective cross section, $I_{\|,\perp} = \int_\mathrm{S} \mathbf{J}\cdot d\mathbf{A}_{\|,\perp}$. Due to spin-orbit coupling, the external field may also induce currents flowing in the transverse direction due to anomalous or planar Hall effects. They are intrinsically included in our results discussed in the main text, but will be neglected in this semi-classical approach since they give higher order terms in the spin-orbit coupling.

The flow of the charge current through the heavy metal generates a spin current with polarization σ flowing in the direction of $\mathbf{J}_\mathrm{S} = \theta_\mathrm{SH}\mathbf{J}_\mathrm{C}\times\sigma$, where θ_SH represents the charge-to-spin conversion rate given by the spin Hall angle. The spin angular momentum that flows along the out-of-plane direction $\hat{z}$ accumulate at the edges of the material, inducing a non-equilibrium spin polarization given by

$$\delta\mathbf{s} \propto \theta_\mathrm{SH}\sigma_0\hat{\mathbf{z}}\times\mathbf{E}\,. \tag{1}$$

The current-induced polarization interacts with the magnetic moment of the ferromagnetic layer, exerting torques that are called spin-orbit torques (SOTs). In general, these torques, to first order in $\delta\mathbf{s}$, have two components[1]: one is odd (also called field-like or reactive torque) with respect to the inversion of the magnetization direction $\mathbf{m}$, and the other is even (called damping-like or dissipative term). They are proportional to $\mathbf{m}\times\delta\mathbf{s}$ and $\mathbf{m}\times(\mathbf{m}\times\delta\mathbf{s})$, respectively, and can be written as

$$\begin{aligned}\tau_\mathrm{odd} &= \left.\frac{d\mathbf{m}}{dt}\right|_\mathrm{odd} = -\gamma\mathbf{m}\times(T_\mathrm{odd}\theta_\mathrm{SH}\sigma_0\hat{\mathbf{z}}\times\mathbf{E})\\ \tau_\mathrm{even} &= \left.\frac{d\mathbf{m}}{dt}\right|_\mathrm{even} = -\gamma\mathbf{m}\times[T_\mathrm{even}\theta_\mathrm{SH}\sigma_0\mathbf{m}\times(\hat{\mathbf{z}}\times\mathbf{E})]\end{aligned} \tag{2}$$

For the ferromagnetic/heavy metal bilayers we study, the inversion symmetry is broken and the torques τ_odd and τ_even have contributions from both spin Hall and inverse spin galvanic effects. In addition, the transverse currents generated by the anomalous and planar Hall effects can also induce torques on the magnetization, which are neglected here.

The magnetization dynamics is affected by both torques, and may be described by the Landau-Lifshitz-Gilbert (LLG) equation

$$\frac{d\mathbf{m}}{dt} = -\gamma\mathbf{m}\times\mathbf{B}^\mathrm{eff} + \alpha\mathbf{m}\times\frac{d\mathbf{m}}{dt} + \tau_\mathrm{odd} + \tau_\mathrm{even} \tag{3}$$

where γ is the gyromagnetic ratio, α denotes the Gilbert damping and the effective magnetic field obtained from the unperturbed hamiltonian, $\mathbf{B}^\mathrm{eff} = \frac{\partial H}{\partial\mathbf{m}}$, includes both the magnetic anisotropy and external magnetic field.

Charge and spin-pumping from magnetization precession

When the electric field is oscillatory, i.e., $\mathbf{E}(t) = \mathrm{Re}\{\mathbf{E}_0 e^{i\omega t}\}$, the spin-orbit torques may set the magnetization into precessional motion[2,3]. The magnetization direction can be written as $\mathbf{m} = \mathbf{m}_0 + \delta\mathbf{m}$, where $\mathbf{m}_0$ is the equilibrium magnetization direction and $\delta\mathbf{m} = \delta\mathbf{m}(t)$ gives the time-dependent deviation from equilibrium. In linear order on the perturbation, the general LLG equation becomes

$$\begin{aligned}\frac{\gamma}{1+\alpha^2}\delta\mathbf{m}\times\mathbf{B}^\mathrm{eff} + \frac{\alpha\gamma B^\mathrm{eff}}{1+\alpha^2}\delta\mathbf{m} + \frac{d\delta\mathbf{m}}{dt}&\\ = -\gamma\frac{(T_\mathrm{odd}-\alpha T_\mathrm{even})\theta_\mathrm{SH}\sigma_0}{1+\alpha^2}&\mathbf{m}_0\times(\hat{\mathbf{z}}\times\mathbf{E})\\ -\gamma\frac{(T_\mathrm{even}+\alpha T_\mathrm{odd})\theta_\mathrm{SH}\sigma_0}{1+\alpha^2}&\mathbf{m}_0\times[\mathbf{m}_0\times(\hat{\mathbf{z}}\times\mathbf{E})]\end{aligned} \tag{4}$$

since $\mathbf{B}^\mathrm{eff}\|\mathbf{m}_0$. Magnetization dynamics induces a flow of angular momentum out of the magnetic unit, a phenomenon called spin pumping. In the bilayers we consider, a spin current is injected from the ferromagnet into the heavy metal flowing in the

out-of-plane $-\hat{\mathbf{z}}$ direction with polarization σ^{pump}, which in linear response is given by[4]

$$\sigma^{\text{pump}} = \frac{1}{4\pi}\left(G_{\text{r}}\mathbf{m}_0 \times \frac{d\delta\mathbf{m}}{dt} + G_{\text{i}}\frac{d\delta\mathbf{m}}{dt}\right) \tag{5}$$

In the equation above, G_{r} and G_{i} are the real and imaginary parts of the spin mixing conductance (related to the interface reflection and transmission amplitudes)[5].

In the presence of spin-orbit coupling, a charge current $\mathbf{J}_{\text{C}}^{\text{dyn}} \propto \theta_{\text{SH}}\hat{\mathbf{z}}\times\sigma$ is generated. Here, σ includes the oscillatory polarization of the spin current σ^{pump} that is converted to charge current through the inverse spin Hall effect and also a contribution from the spin galvanic effect (which, in this context, is also called magnonic charge pumping[6]).

Taking into account that for sufficiently low fields the solution of Eq. 4 is a vector $\delta\mathbf{m}$ that lies in the plane perpendicular to $\mathbf{m}_0$, it can be written in the basis formed by the vectors $\mathbf{m}_0\times(\hat{\mathbf{z}}\times\mathbf{E})$ and $\mathbf{m}_0\times[\mathbf{m}_0\times(\hat{\mathbf{z}}\times\mathbf{E})]$. Therefore, the charge current generated by the magnetization dynamics can be written as

$$\begin{aligned}\mathbf{J}_{\text{C}}^{\text{dyn}}(t) = \theta_{\text{SH}}^2\sigma_0 \Big\{&\sigma_2^{\text{dyn}}(\omega)(\mathbf{m}_0\cdot\hat{\mathbf{z}})(\hat{\mathbf{z}}\times\mathbf{E}) \\ &+\sigma_1^{\text{dyn}}(\omega)\left[(\mathbf{m}_0-(\mathbf{m}_0\cdot\hat{\mathbf{z}})\hat{\mathbf{z}})(\mathbf{m}_0\cdot\mathbf{E})+(\mathbf{m}_0\cdot\hat{\mathbf{z}})^2\mathbf{E}\right]\Big\}\end{aligned} \tag{6}$$

Equation 2 of the main text is obtained by integrating the equation above over cross sections of the longitudinal and transverse directions. The spin Hall magnetoresistance contributions for static perturbations have the same symmetry as the ones obtained above (see Eqs. 19 and 20 of Ref.[7]). Nevertheless, they are not included in the equation above since, for static magnetizations, $\frac{d\mathbf{m}}{dt}=0$ and no current is pumped. It can be obtained, however, by substituting $\frac{d\mathbf{m}}{dt}$ of Eq. 5 by the static torque $\mathbf{m}\times\delta\mathbf{s}$. Similar results were found in Ref. 8.

S2 Formalism

In this section, we detail the quantum formalism used to obtain the results of the main text. The system is described by the hamiltonian

$$\hat{H} = \hat{H}_0 + \hat{H}_{\text{int}} + \hat{H}_{\text{Z}} + \hat{H}_{\text{so}} \ . \tag{7}$$

Here, $\hat{H}_0$ is a multi-orbital tight-binding hamiltonian that includes nine orbitals per atom (s, p and d) and is given by

$$\hat{H}_0 = \sum_{\substack{mn\\s}}\sum_{\alpha\beta} t_{mn}^{\alpha\beta} c_{m\alpha s}^{\dagger} c_{n\beta s} \ , \tag{8}$$

where $c_{m\alpha s}^{\dagger}$ ($c_{m\alpha s}$) is the creation (annihilation) operator of an electron with spin s on orbital α of site m, and the hopping matrices $t_{mn}^{\alpha\beta}$ are obtained from paramagnetic first principles calculations[9]. The screened electron-electron interaction is included through the Hubbard-like hamiltonian

$$\hat{H}_{\text{int}} = \frac{1}{2}\sum_{\substack{m\\s,s'}}\sum_{\alpha\beta\gamma\xi} U_{\alpha\beta\gamma\xi}^{m} c_{m\alpha s}^{\dagger} c_{m\beta s'}^{\dagger} c_{m\xi s'} c_{m\gamma s}, \tag{9}$$

where the screened interaction is taken to be local and given by

$$U_{\alpha\beta\gamma\xi}^{m} = \iint d\mathbf{r}d\mathbf{r}'\phi_{\alpha}^{*}(\mathbf{r}-\mathbf{R}_m)\phi_{\beta}^{*}(\mathbf{r}'-\mathbf{R}_m)\frac{e}{|\mathbf{r}-\mathbf{r}'|}\phi_{\gamma}(\mathbf{r}-\mathbf{R}_m)\phi_{\xi}(\mathbf{r}'-\mathbf{R}_m) = U_{\beta\alpha\xi\gamma}^{m} \ . \tag{10}$$

We treat the effective Coulomb interaction matrix on site m, given by $U_{\alpha\beta\gamma\xi}^{m}$, in the Lowde-Windsor approximation[10], i.e., $U_{\alpha\beta\gamma\xi}^{m} = U^m\delta_{\alpha\xi}\delta_{\beta\gamma}$, with $U^m = 1$eV for the d orbitals of both the ferromagnetic and heavy metal layers, and zero for the sp complex. The external static magnetic field is included through the spin and orbital Zeeman interaction hamiltonians

$$\hat{H}_{\text{Z}} = \mu_B\sum_m (g_{\text{S}}\hat{\mathbf{S}}_m + g_{\text{L}}\hat{\mathbf{L}}_m)\cdot\mathbf{B} \ , \tag{11}$$

where μ_B is the Bohr magneton, g_{S} and g_{L} are the spin and orbital Landé g-factors. Finally, we include the spin-orbit interaction using the local hamiltonian

$$\hat{H}_{\text{so}} = \sum_m \lambda_m\hat{\mathbf{L}}_m\cdot\hat{\mathbf{S}}_m \ . \tag{12}$$

The spin-orbit parameter λ_m is also obtained from first principles calculations.

We start from the paramagnetic system described by Eq. 8 and include the electronic interaction given in Eq. 9 in the mean-field approximation to generate the spin-polarized one-electron ground state. This is done self-consistently by assuming a fixed value of the Fermi energy and adjusting the atomic d-orbital energy levels to reproduce the electronic occupancy obtained by the RS-LMTO-ASA calculations for each layer[11].

The excitations of the system are investigated through the response functions obtained using linear response theory[12]. We define a generalized dynamical susceptibility in real space as

$$\chi_{ijkl}^{\alpha\beta\,\mu\nu\gamma\xi}(t) = -\frac{i}{4\hbar}\Theta(t)\sum_{\substack{s_1s_2\\s_3s_4}}\langle[c_{i\mu s_1}^{\dagger}(t)\sigma_{s_1s_2}^{\alpha}c_{j\nu s_2}(t), c_{k\gamma s_3}^{\dagger}\sigma_{s_3s_4}^{\beta}c_{l\xi s_4}]\rangle \ , \tag{13}$$

where i,j,k,l are site indices, μ,ν,γ,ξ denotes the orbitals, and α,β are the spin components described by the Pauli matrix σ. All the responses we calculate are written in terms of the generalized susceptibility.

When a time-dependent uniform external electric field $\mathbf{E}(\mathbf{r},t) = \mathrm{Re}\{E_0\hat{\mathbf{x}}e^{i\omega t}\}$ is applied to a bilayer system, the interaction hamiltonian can be written as

$$\begin{aligned}\hat{H}_{\mathrm{pert}}(t) &= -\int d\mathbf{r}\hat{\mathbf{J}}^{\mathrm{C}}(\mathbf{r},t)\cdot\mathbf{A}(\mathbf{r},t)\\ &= \frac{eE_0}{\hbar\omega}\frac{1}{N_{\parallel}}\sum_{\mathbf{k}_{\parallel},\sigma}\sum_{\substack{\ell\ell'\\\mu\nu}}\nabla_{\mathbf{k}_{\parallel}}t_{\ell\ell'}^{\mu\nu}(\mathbf{k}_{\parallel})\cdot\hat{\mathbf{x}}\ \sin(\omega t)c_{\ell\mu\sigma}^{\dagger}(\mathbf{k}_{\parallel},t)c_{\ell'\nu\sigma}(\mathbf{k}_{\parallel},t) \ ,\end{aligned} \tag{14}$$

where ℓ and ℓ' denote plane indices and $\mathbf{k}_{\parallel}$ is an in-plane wave vector inside the first Brillouin Zone that is summed over $N_{\parallel}$ points. Within linear response theory, the charge current flowing through a layered system, from site $\mathbf{R}_{\parallel}$ to $\mathbf{R}'_{\parallel}$ on the same layer ℓ_1, can be written as

$$\begin{aligned}\langle\hat{I}_{\ell_1}^{\mathrm{C}}(\mathbf{R}_{\parallel},\mathbf{R}'_{\parallel})\rangle &= -\frac{e^2}{\hbar}\mathrm{Re}\left\{\frac{e^{-i\omega t}}{\hbar\omega}\mathscr{I}_{\ell_1}^{\mathrm{C}}(\mathbf{R}'_{\parallel}-\mathbf{R}_{\parallel},\omega)\right\}E_0 + \langle\hat{I}_{\ell_1}^{\mathrm{dia}}(\mathbf{R}_{\parallel},\mathbf{R}'_{\parallel})\rangle\\ &= -\frac{e^2}{\hbar^2\omega}|\mathscr{I}_{\ell_1}^{\mathrm{C}}(\mathbf{R}'_{\parallel}-\mathbf{R}_{\parallel},\omega)|E_0\cos[\omega t-\phi^{\mathrm{C}}(\omega)] + \langle\hat{I}_{\ell_1}^{\mathrm{dia}}(\mathbf{R}_{\parallel},\mathbf{R}'_{\parallel})\rangle \ .\end{aligned} \tag{15}$$

where e is the electronic charge, $\hbar$ is the Planck's constant, and the absolute value of $\mathscr{I}_{\ell_1}^{\mathrm{C}}(\mathbf{R}'_{\parallel}-\mathbf{R}_{\parallel},\omega)$ and its phase $\phi^{\mathrm{C}}(\omega)$ are given in terms of the Fourier transform of the generalized susceptibility defined in Eq. 13 as

$$\begin{aligned}\mathscr{I}_{\ell_1}^{\mathrm{C}}(\mathbf{R}'_{\parallel}-\mathbf{R}_{\parallel},\omega) = &\frac{1}{N_{\parallel}}\sum_{\substack{\mathbf{k}_{\parallel}\\\sigma\sigma'}}\sum_{\substack{\ell\ell'\\\mu\nu\gamma\xi}}\left[t_{\ell_1\ell_1}^{\mu\nu}(\mathbf{R}'_{\parallel}-\mathbf{R}_{\parallel})e^{i\mathbf{k}_{\parallel}\cdot(\mathbf{R}'_{\parallel}-\mathbf{R}_{\parallel})} - t_{\ell_1\ell_1}^{\nu\mu}(\mathbf{R}'_{\parallel}-\mathbf{R}_{\parallel})e^{-i\mathbf{k}_{\parallel}\cdot(\mathbf{R}'_{\parallel}-\mathbf{R}_{\parallel})}\right]\\ &\times\chi_{\ell_1\ell_1\ell\ell'}^{\sigma\sigma'\,\mu\nu\gamma\xi}(\mathbf{k}_{\parallel},\mathbf{k}_{\parallel},\omega)\nabla_{\mathbf{k}_{\parallel}}t_{\ell\ell'}^{\gamma\xi}(\mathbf{k}_{\parallel})\cdot\hat{\mathbf{x}} \ .\end{aligned} \tag{16}$$

Here, σ,σ' are spin indices that are summed over $\uparrow$ and $\downarrow$ states, ℓ,ℓ' are plane indices covering the whole system, and μ,ν,γ,ξ are orbital indices. The diamagnetic current $\langle\hat{I}_{\ell_1}^{\mathrm{dia}}(\mathbf{R}_{\parallel},\mathbf{R}'_{\parallel})\rangle$ on Eq. 15 is a ground state contribution along the longitudinal direction that can be written as $C_{\ell_1}(\mathbf{R}_{\parallel},\mathbf{R}'_{\parallel})\sin(\omega t)/\omega$. The value of $C_{\ell_1}(\mathbf{R}_{\parallel},\mathbf{R}'_{\parallel})$ is obtained using the f-sum rule[13,14], such that $\mathscr{I}_{\ell_1}^{\mathrm{C}}(\mathbf{R}'_{\parallel}-\mathbf{R}_{\parallel},\omega=0)+C_{\ell_1}(\mathbf{R}_{\parallel},\mathbf{R}'_{\parallel})=0$.

The total longitudinal and transverse currents per electric field, $I_{\omega,\parallel}$ and $I_{\omega,\perp}$, described in the main text, are obtained by summing

$$\begin{aligned}I_{\omega,\parallel} &= \sum_{\ell_1}\sum_{\mathbf{R}'}\langle\hat{I}_{\ell_1}^{\mathrm{C}}(\mathbf{R}_{\parallel},\mathbf{R}'_{\parallel})\rangle\hat{\mathbf{r}}\cdot\hat{\mathbf{x}}\\ I_{\omega,\perp} &= \sum_{\ell_1}\sum_{\mathbf{R}'}\langle\hat{I}_{\ell_1}^{\mathrm{C}}(\mathbf{R}_{\parallel},\mathbf{R}'_{\parallel})\rangle\hat{\mathbf{r}}\cdot\hat{\mathbf{y}}\end{aligned} \ , \tag{17}$$

where $\hat{\mathbf{r}}$ is a unit vector along the direction of the bond, $\mathbf{R}'_{\parallel}-\mathbf{R}_{\parallel}$, and the sum over $\mathbf{R}'_{\parallel}$ covers sites with positive x (y) component for the longitudinal (transverse) current. Our results are given in units of $(e^2/\hbar)$Å.

Within the RPA, the elements of the generalized susceptibility can be expressed in terms of the noninteracting spin susceptibilities χ_0 that are generated by evaluating the commutators of Eq. 13 in the noninteracting ground state. In matrix form the relation is schematically given

$$[\chi] = [\chi_0] - [\chi_0][U][\chi] \ . \tag{18}$$

In particular, the elements of its Fourier transform can be obtained by

$$\chi^{\mu\nu\gamma\xi}_{iik\ell}(\mathbf{k}_\parallel,\mathbf{k}_\parallel;\omega) = \sum_{\substack{m\\ \alpha\beta}} [1+\chi_0(0;\omega)U]^{-1\,\mu\nu\alpha\beta}_{iimm}\, \chi^{\alpha\beta\gamma\xi}_{0mmk\ell}(\mathbf{k}_\parallel,\mathbf{k}_\parallel;\omega)\ . \tag{19}$$

Here, χ and U are 4×4 matrices in the spin space that covers charge density ρ and the spin density components S^x, S^y, S^z. These matrices can also be written in the basis $S^+, S^\uparrow, S^\downarrow, S^-$. $\chi_0(0;\omega)$ is the usual (non-generalized) susceptibility calculated for $\mathbf{q}=0$. The U matrix is obtained from the equation of motion of the susceptibilities using the hamiltonian $\hat{H}_{\text{int}}$ written in Eq. 9. It can be written as

$$[U]^{\alpha\beta\gamma\xi}_{mnkl} = \begin{pmatrix} U^m_{\alpha\gamma\xi\beta} & 0 & 0 & 0 \\ 0 & U^m_{\alpha\gamma\xi\beta} - U^m_{\gamma\alpha\xi\beta} & -U^m_{\gamma\alpha\xi\beta} & 0 \\ 0 & -U^m_{\gamma\alpha\xi\beta} & U^m_{\alpha\gamma\xi\beta} - U^m_{\gamma\alpha\xi\beta} & 0 \\ 0 & 0 & 0 & U^m_{\alpha\gamma\xi\beta} \end{pmatrix} \delta_{km}\delta_{lm}\delta_{mn} \quad . \tag{20}$$

The mean-field susceptibility χ_0 is calculated in terms of single-particle Green functions as

$$\begin{aligned}\chi^{\alpha\beta\,\mu\nu\gamma\xi}_{0ijk\ell}(\mathbf{k}_1,\mathbf{k}_2;\omega) = -\frac{\hbar}{4\pi}\int d\omega' f(\omega')\mathrm{Tr}\Big\{&\sigma^\alpha G^{\nu\gamma}_{jk}(\mathbf{k}_1;\omega'+\omega+i\eta)\sigma^\beta \Im\mathrm{m}[G^{\xi\mu}_{\ell i}(\mathbf{k}_2;\omega')] \\ &+\sigma^\alpha \Im\mathrm{m}[G^{\nu\gamma}_{jk}(\mathbf{k}_1;\omega')]\sigma^\beta G^{\xi\mu}_{\ell i}(\mathbf{k}_2;\omega'-\omega-i\eta)\Big\}\ .\end{aligned} \tag{21}$$

where $i\eta$ is a small imaginary part added to the energy in order to shift the poles of the Green functions from the real axis, that sometimes is used to mimic the effect of disorder[15,16]. We have defined $\Im\mathrm{m}[G(\omega)] = [G(\omega+i\eta)-G(\omega-i\eta)]/2i$.

S3 Ground state properties

The sample composed by a monolayer of Co deposited on four layers of Pt(001) presents an out-of-plane easy-axis, which can be mapped into a simple model with energy given by $E=-K_z\cos^2\theta - \mathbf{m}\cdot\mathbf{B}^{\text{ext}}$. From the static transverse magnetic susceptibility[17], we obtain an anisotropy of $K_z \simeq 1.17\,\text{meV}$. When no magnetic field is applied, the magnetization on the Co layer is $\mathbf{m}_{\text{Co}} \sim 1.7\mu_B\hat{\mathbf{z}}$, and the Pt layers next to the interface also acquire a small spin polarization due to the proximity effect. For example, the Pt layer at the interface with the Co layer has a magnetization of $\mathbf{m}_{\text{Pt}_1} \sim 0.24\mu_B\hat{\mathbf{z}}$. To obtain the angular dependencies shown in Fig. 3(a,b) and Fig. 4 of the main text, we use a magnetic field defined by the Zeeman energy $g_S\mu_B B^{\text{ext}} \simeq 4.1\,\text{meV}$. Such large magnetic fields values are used to overcome the relatively strong anisotropies in our ultrathin films.

The Fe layer deposited on top of four W(110) layers presents a magnetization of $\mathbf{m}_{\text{Fe}} \sim 2.3\mu_B\hat{\mathbf{x}}$, with an induced magnetization in the W interface layer of $\mathbf{m}_{\text{W}_1} \sim -0.1\mu_B\hat{\mathbf{x}}$, where $\hat{\mathbf{x}}$ is the direction of the long axis. This system presents biaxial anisotropy which can be mapped in a model with energy given by $E=-K_x\sin^2\theta\cos^2\phi - K_y\sin^2\theta\sin^2\phi - \mathbf{m}\cdot\mathbf{B}^{\text{ext}}$. From the static transverse magnetic susceptibility matrix, we obtain the values $K_x \simeq 2.35\,\text{meV}$ and $K_y \simeq 0.33\,\text{meV}$. The field used to drag the magnetization on Fig. 3(c,d) of the main text is given by the Zeeman energy $g_S\mu_B B^{\text{ext}} \simeq 6.8\,\text{meV}$.

S4 Feedback of magnetization dynamics on the charge currents

In the main text, we have demonstrated that the current-induced magnetization dynamics generates a feedback signal in the charge currents. The relation between these quantities can also be seen by analyzing the schematic relation between the response functions within RPA, given by Eq 18, neglecting the longitudinal part of $[U]$ (our calculations, however, include all contributions). It can be used to obtain the response of the current density due to an applied electric field (where the perturbation is given by Eq. 14), which we call, for simplicity, $\chi^{\mathbf{jj}}$. The longitudinal and transverse currents per electric field calculated in the main text are related to $\chi^{\mathbf{jj}}$ by an integral over the respective cross-section. The response $\chi^{\mathbf{jj}}$ is given by

$$[\chi^{\mathbf{jj}}] = [\chi_0^{\mathbf{jj}}] - [\chi_0^{\mathbf{jm}}][U][\chi^{\mathbf{mj}}]\ . \tag{22}$$

$\chi^{\mathbf{mj}}$ is a mixed response function representing the magnetization response to an electric field, and was investigated in Ref. 3; $\chi_0^{\mathbf{jm}}$ is the non-interacting current density response due to magnetic fields (that couples to the magnetization). The responses $\chi^{\mathbf{jm}}$ and $\chi^{\mathbf{mj}}$ are related by Onsager reciprocity. All matrices χ are complex. The symmetries of these quantities are determined by the processes allowed by the spin-orbit coupling and by the lattice structure[6]. Equation 18 can also be particularized for $\chi^{\mathbf{mj}}$, which is then given by

$$[\chi^{\mathbf{mj}}] = [\chi_0^{\mathbf{mj}}] - [\chi^{\mathbf{mm}}][U][\chi_0^{\mathbf{mj}}]\ . \tag{23}$$

This equation exhibits how these responses are connected to the magnetic susceptibility $\chi^{\mathbf{mm}}$. Substituting Eq. 23 back into Eq. 23, we get

$$[\chi^{\mathbf{jj}}] = [\chi_0^{\mathbf{jj}}] - [\chi_0^{\mathbf{jm}}][U]\left(1 - [\chi^{\mathbf{mm}}][U]\right)[\chi_0^{\mathbf{mj}}] \ . \tag{24}$$

This result elucidate how the total charge current is generated. The contribution associated with $\chi_0^{\mathbf{jj}}$ is the current directly driven by the electric field. The second part involves the combination of processes described in the main text: first, a generation of spin polarization described by $\chi_0^{\mathbf{mj}}$; This accumulation couples to the magnetization response inducing its precessional motion described by $\chi^{\mathbf{mm}}$; The magnetization precession is then converted back to charge current by the reciprocal phenomena, symbolized by $\chi_0^{\mathbf{mj}}$. Equation 24 demonstrates that the in-phase and out-of-phase currents involve different combinations of the imaginary part (symmetric Lorentzian) and real part (antisymmetric Lorentzian) of the transverse magnetic susceptibility.

Analogously, Eq. 22 makes clear the relation between the charge currents and the current-induced magnetization precession described by $\chi^{\mathbf{mj}}$. The dynamical contribution (i.e., the second term on the right hand side of the equation) involves a combination of in-phase (real) and out-of-phase (imaginary) parts of the oscillatory magnetization components. Figure **S3** illustrates one example using the results of Fig. 3d of the main text to compare the dynamical contribution to the current with the out-of-phase part of the corresponding magnetization component.

S5 Higher harmonics

There are two main experimental techniques to measure spin-orbit torques or the associated effective fields. The first one measures the rectified (dc) current generated by the frequency mixing effect[2,6,18–22], a static voltage generated when an ac-current excites the magnetization precession. The second technique mesures the signal with twice the frequency of the applied perturbation (usually, very low frequencies), i.e., the second harmonic signal[1,23–26].

Both effects rely on second order signals with respect to the applied perturbation. They can be described taking into account that, in the presence of spin-orbit coupling, the resistance R depends on the magnetization direction. This magnetization, in turn, also depends on the applied currents due to the the spin-orbit effects described in the main text. Therefore, the voltage V measured when a current I is injected in the system can be written as

$$V(I) = R(\mathbf{m}(I))I \quad . \tag{25}$$

In our work, we consider a time-dependent electric field $\mathbf{E}$ that generates an electric current flowing across the system. For a uniform electric field, $E(t) = V(t)/d$ applied along the $\hat{\mathbf{x}}$, we can write the inverse of Eq. 25 as

$$I(E) = G(\mathbf{m}(E))E \quad , \tag{26}$$

where $G(\mathbf{m}(E)) = G'(\mathbf{m}(E))d$ is a measure of the conductance G' of the system. The quantity G is calculated using Eq. 15. Here, the dependency of the magnetization $\mathbf{m}$ on the electric field E, which drives its precession, is made explicit. Expanding the current in powers of E,

$$I(E) = \left.\frac{dI}{dE}\right|_{E=0} E + \frac{1}{2}\left.\frac{d^2I}{dE^2}\right|_{E=0} E^2 + \cdots \frac{1}{n!}\left.\frac{d^nI}{dE^n}\right|_{E=0} E^n \quad . \tag{27}$$

With the use of Eq. 26, we can obtain the derivatives of the current with respect to the electric field as

$$\begin{aligned} \left.\frac{dI}{dE}\right|_{E=0} &= G(\mathbf{m}) \\ \left.\frac{d^2I}{dE^2}\right|_{E=0} &= 2\left.\frac{dG(\mathbf{m}(E))}{dE}\right|_{E=0} \quad , \\ &\cdots \\ \left.\frac{d^nI}{dE^n}\right|_{E=0} &= n\left.\frac{d^{n-1}G(\mathbf{m}(E))}{dE^{n-1}}\right|_{E=0} \end{aligned} \tag{28}$$

If we consider a harmonic field given by $\mathbf{E}(t) = E_0\cos(\omega t)\hat{\mathbf{x}}$, the first and second order terms are given by

$$\begin{aligned} I^{(1)}(E) &= G(\mathbf{m})E \\ I^{(2)}(E) &= \left.\frac{dG(\mathbf{m}(E))}{dE}\right|_{E=0} E_0^2\cos^2(\omega t) \quad . \\ &= \left[\frac{dG(\mathbf{m})}{dm^x}\chi^{m^x j} + \frac{dG(\mathbf{m})}{dm^y}\chi^{m^y j} + \frac{dG(\mathbf{m})}{dm^z}\chi^{m^z j}\right]\frac{E_0^2}{2}\left[1+\cos(2\omega t)\right] \end{aligned} \tag{29}$$

We have identified the derivatives of the magnetization with respect to the electric field with the mixed response functions $\chi^{\mathbf{m}j}$ described in Sec. S2. These responses are usually obtained through the solution of the generalized LLG equation showed in Sec. S1 (see, for example, Refs. 2,6,22 and references therein), while the derivatives of the conductance (or resistance) with respect to the magnetization components are obtained from the usual magnetoresistance and Hall currents formulas. As Eq. 29 shows, the second order term for the dc current and the second harmonic $\cos(2\omega t)$ have the same origin and are given by the same coefficients.

To understand how the effects discussed in the main text can affect the second order signals, we consider the longitudinal currents described by the usual anisotropic magnetoresistance (AMR) and spin Hall magnetoresistance (SMR). For relatively small magnetoresistance ratios ($\Delta R \ll R_0$), the linear term of the longitudinal current can be written as

$$I^{(1)}(E) = \left[G_0 + \Delta G_{\mathrm{AMR}} \left(m_0^x\right)^2 + \Delta G_{\mathrm{SMR}} \left(m_0^y\right)^2\right] E\cos(\omega t)\ . \tag{30}$$

This is the current explored on the main text, in which we show that ΔG depends drastically on the frequency of the electric field and on the intensity of the magnetic field.

In this case, the second order longitudinal currents are given by

$$I^{(2)}(E) = \left[\Delta G_{\mathrm{AMR}}(\omega,|\mathbf{B}|) m_0^x \chi^{m^x j}(\omega,|\mathbf{B}|) + \Delta G_{\mathrm{SMR}}(\omega,|\mathbf{B}|) m_0^y \chi^{m^y j}(\omega,|\mathbf{B}|)\right] E_0^2 \left[1+\cos(2\omega t)\right] \tag{31}$$

Here, we have explicitly included the dependencies of both linear response functions $\Delta G(\omega,|\mathbf{B}|)$ and $\chi^{\mathbf{m}j}(\omega,|\mathbf{B}|)$ on the frequency of the electric field and intensity of the magnetic field. This means that these dependencies originate not only from the magnetic responses, but also from the conductance/resistance. Since second harmonic measurements are obtained on very low frequencies, the variations of ΔG are small (as seen in Fig. 1(a-c) of the main text). Nevertheless, experiments that varies the frequency or the magnetic field through the ferromagnetic resonance[2,6,18–22] may incur in deviations of the measured quantities by considering ΔG (or ΔR) as a constant.

References

1. Garello, K. *et al.* Symmetry and magnitude of spin-orbit torques in ferromagnetic heterostructures. *Nature Nanotechnol.* **8**, 587–593 (2013).
2. Fang, D. *et al.* Spin–orbit-driven ferromagnetic resonance. *Nature Nanotechnol.* **6**, 413–417 (2011).
3. Guimarães, F. S. M., Lounis, S., Costa, A. T. & Muniz, R. B. Dynamical current-induced ferromagnetic and antiferromagnetic resonances. *Phys. Rev. B* **92**, 220410(R) (2015).
4. Tserkovnyak, Y., Brataas, A. & Bauer, G. E. W. Enhanced Gilbert Damping in Thin Ferromagnetic Films. *Phys. Rev. Lett.* **88**, 117601 (2002).
5. Tserkovnyak, Y., Brataas, A., Bauer, G. E. W. & Halperin, B. Nonlocal magnetization dynamics in ferromagnetic heterostructures. *Rev. Mod. Phys.* (2005).
6. Ciccarelli, C. *et al.* Magnonic charge pumping via spin–orbit coupling. *Nature Nanotechnol.* **10**, 50–54 (2014).
7. Chen, Y.-T. *et al.* Theory of spin Hall magnetoresistance. *Phys. Rev. B* **87**, 144411 (2013).
8. Tserkovnyak, Y. & Bender, S. A. Spin Hall phenomenology of magnetic dynamics. *Phys. Rev. B* **90**, 014428 (2014).
9. Andersen, O. K. & Jepsen, O. Explicit, First-Principles Tight-Binding Theory. *Phys. Rev. Lett.* **53**, 2571–2574 (1984).
10. Lowde, R. D. & Windsor, C. G. On the magnetic excitations in nickel. *Advances in Physics* **19**, 813–909 (2006).
11. Muniz, R. B. & Mills, D. L. Theory of spin excitations in Fe(110) monolayers. *Phys. Rev. B* **66**, 174417 (2002).
12. Kubo, R. Statistical-Mechanical Theory of Irreversible Processes. I. General Theory and Simple Applications to Magnetic and Conduction Problems. *J. Phys. Soc. Jpn.* **12**, 570–586 (1957).
13. Benfatto, L., Sharapov, S. G., Andrenacci, N. & Beck, H. Ward identity and optical conductivity sum rule in the *d*-density wave state. *Phys. Rev. B* **71**, 104511 (2005).
14. Raimbault, N., de Boeij, P. L., Romaniello, P. & Berger, J. A. Gauge-Invariant Calculation of Static and Dynamical Magnetic Properties from the Current Density. *Phys. Rev. Lett.* **114**, 066404 (2015).
15. Freimuth, F., Blügel, S. & Mokrousov, Y. Spin-orbit torques in Co/Pt(111) and Mn/W(001) magnetic bilayers from first principles. *Phys. Rev. B* **90**, 174423 (2014).
16. Freimuth, F., Blügel, S. & Mokrousov, Y. Direct and inverse spin-orbit torques. *Phys. Rev. B* **92**, 064415 (2015).

17. dos Santos Dias, M., Schweflinghaus, B., Blügel, S. & Lounis, S. Relativistic dynamical spin excitations of magnetic adatoms. *Phys. Rev. B* **91**, 075405 (2015).

18. Yamaguchi, A. *et al.* Broadband ferromagnetic resonance of $Ni_{81}Fe_{19}$ wires using a rectifying effect. *Phys. Rev. B* **78**, 104401 (2008).

19. Mosendz, O. *et al.* Quantifying Spin Hall Angles from Spin Pumping: Experiments and Theory. *Phys. Rev. Lett.* **104**, 046601 (2010).

20. Liu, L., Moriyama, T., Ralph, D. C. & Buhrman, R. A. Spin-Torque Ferromagnetic Resonance Induced by the Spin Hall Effect. *Phys. Rev. Lett.* **106**, 036601 (2011).

21. Liu, L. *et al.* Spin-Torque Switching with the Giant Spin Hall Effect of Tantalum. *Science* **336**, 555–558 (2012).

22. Kurebayashi, H. *et al.* An antidamping spin-orbit torque originating from the Berry curvature. *Nature Nanotechnol.* **9**, 211–217 (2014).

23. Hayashi, M., Kim, J., Yamanouchi, M. & Ohno, H. Quantitative characterization of the spin-orbit torque using harmonic Hall voltage measurements. *Phys. Rev. B* **89**, 144425 (2014).

24. Avci, C. O. *et al.* Fieldlike and antidamping spin-orbit torques in as-grown and annealed Ta/CoFeB/MgO layers. *Phys. Rev. B* **89**, 214419 (2014).

25. Avci, C. O. *et al.* Unidirectional spin Hall magnetoresistance in ferromagnet/normal metal bilayers. *Nature Phys.* **11**, 570–575 (2015).

26. Reichlová, H. *et al.* Current-induced torques in structures with ultrathin IrMn antiferromagnets. *Phys. Rev. B* **92**, 165424 (2015).
